# Antegrade Auriculotemporal-Inferior Alveolar Nerve Communication in the Infratemporal Fossa

**DOI:** 10.7759/cureus.50526

**Published:** 2023-12-14

**Authors:** Tara Tritsch, Mohammadali M Shoja, R. Shane Tubbs

**Affiliations:** 1 Medical Education, Nova Southeastern University, Fort Lauderdale, USA; 2 Anatomical Sciences, St. George's University, St. George's, GRD; 3 Neurosurgery and Structural & Cellular Biology, Tulane University School of Medicine, New Orleans, USA; 4 Neurosurgery and Ochsner Neuroscience, Ochsner Health System, New Orleans, USA

**Keywords:** inferior alveolar nerve, cranial nerve, nerve anastomosis, infratemporal fossa, auriculotemporal nerve

## Abstract

Communications between cranial nerves or their branches have been described previously. The exact functional significance of some of these neural communications remains to be fully understood. This paper reports a unique communication between the auriculotemporal and inferior alveolar nerves within the infratemporal fossa. The histological examination indicates an antegrade connection from the inferior alveolar nerve to the auriculotemporal nerve, which could potentially be implicated in referred pain from the anatomical territory of one nerve to the other.

## Introduction

The infratemporal fossa, situated immediately medial to the mandibular ramus, serves as a central hub for the distribution of the mandibular division of the trigeminal nerve. The four main branches of the mandibular nerve are lingual, inferior alveolar, long buccal, and auriculotemporal nerves, with the latter carrying postsynaptic parasympathetic fibers from the otic ganglion. The auriculotemporal nerve exhibits notable variability, including the presence of single, double, triple, or even quadruple roots [1,2]. The nerve may display a connecting branch between its upper and lower roots, may have between five to nine terminal branches, and may fenestrate the superficial temporal vein [1,3]. In some cases, it can also establish connections with adjacent nerves, such as the lesser occipital nerve temporofacial division or temporal branch of the facial nerve [1,4]. The nerve occasionally communicates with other branches of the trigeminal nerve, notably the zygomaticotemporal branch of the zygomatic branch of the maxillary nerve [5]. The precise functional significance of auriculotemporal nerve communications remains incompletely understood [4]. One study has observed that the fibers within the communicating auriculotemporal nerves in facial nerve branches consistently innervated certain upper muscles of facial expression [6]. In this report, we examine an instance of neural communication between the auriculotemporal and inferior alveolar nerves through both gross and histological studies.

## Case presentation

**Figure 1:**
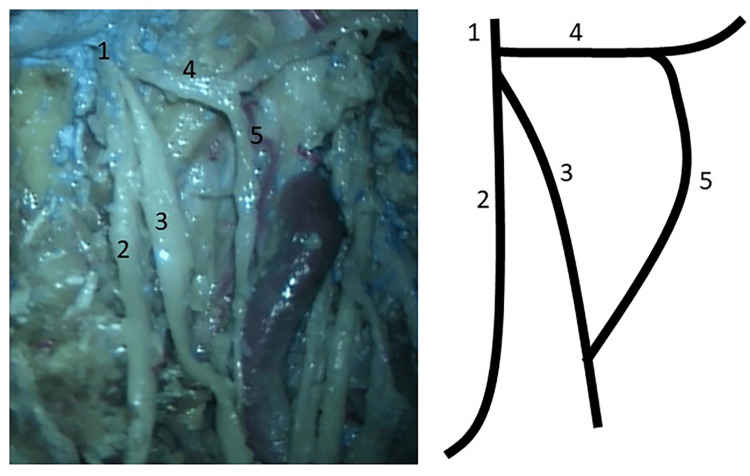
A view of the infratemporal fossa after the removal of superficial structures. Note the mandibular nerve (1) gives rise to the lingual (2), inferior alveolar (3), and auriculotemporal nerves (4). While the first two nerves course anteroinferiorly, the auriculotemporal nerve initially courses backward and horizontally in the infratemporal fossa. Note the communication between auriculotemporal and inferior alveolar nerves (5). A schema is shown on the right side of the picture.

During dissection of the infratemporal fossa in a formalin-fixed, latex-injected, adult head specimen, a neural communication was found between the auriculotemporal and inferior alveolar nerves. Briefly, after the removal of the masseter muscle and outer cortex of the mandible, the inferior alveolar nerve and artery were isolated and traced to their origin. The condyle and coronoid process of the mandible and lateral pterygoid muscles were removed. The lingual nerve was located and traced to its origin from the otic ganglion. Next, the middle meningeal artery and its surrounding auriculotemporal nerve fibers were isolated. It was noted that a branch from the auriculotemporal nerve traveled anteroinferiorly over the medial pterygoid muscle to join the inferior alveolar nerve in the infratemporal fossa (Figure 1). Specimens from this communicating branch and auriculotemporal nerve before and after this communication were fixed in 10% buffered formalin solution and submitted for histological examination using Luxol fast blue with hematoxylin-eosin staining. On histology, the communicating branch contained 1 nerve fascicle without ganglion cells. Before communicating, the auriculotemporal nerve had approximately 12 nerve fascicles with a cluster of ganglion cells. The portion of the auriculotemporal nerve distal to the communication had 10 nerve fascicles with no ganglion cells.

## Discussion

The prevalence of neural communications between the auriculotemporal and inferior alveolar nerves varies in the literature, likely owing to the incidental nature of this observation and the limited number of specimens examined. Anecdotally, we have rarely observed this anastomosis during infratemporal dissections. Anil and colleagues noted the bilateral occurrence of communication between the auriculotemporal and inferior alveolar nerves in one out of 10 specimens they dissected [7]. The second part of the maxillary artery passes through the loop formed by the communicating branch of the auriculotemporal nerve and the mandibular nerve proximal to the origin of the inferior alveolar nerve. Gülekon and colleagues also observed a connecting branch between the auriculotemporal and inferior alveolar nerves in four out of 32 infratemporal fossae [2]. Buch and Agnihotri reported a recurrent branch of the inferior alveolar nerve, which existed in approximately 45% of cadavers [8]; in one of the cases they described, the recurrent branch bifurcated, the two divisions enclosed the maxillary artery and then joined the mandibular nerve. There was a communication between a division of the recurrent nerve and the auriculotemporal nerve.

The present case shows some differences from those reported before. First, the maxillary artery did not pass through the loop formed by the communicating branch between the auriculotemporal and inferior alveolar nerves. Second, on histological examination, the auriculotemporal nerve, before and after the communicating branch, had 12 and 10 nerve fascicles, respectively. Given that both the auriculotemporal and inferior alveolar nerves are primarily sensory in function, this finding suggests the possibility of sensory fibers traveling from the inferior alveolar nerve to the auriculotemporal nerve. Last, we noted that the auriculotemporal nerve contained several ganglion cells. Whether these ganglions represent sensory or secretomotor neurons is not clear and should be investigated in future studies. Ganglion cells have been reported in the trunk of several other cranial nerves (e.g., glossopharyngeal, spinal accessory, and hypoglossal nerves) and their branches [9-11]. The neural communication between the auriculotemporal and inferior alveolar nerves is clinically significant as, for example, pain can be referred from the anatomical territory of one nerve to the other.

## Conclusions

Occasional neural communications exist between the branches of the mandibular nerve within the infratemporal fossa. The functional significance of these interconnections is yet to be fully understood. In this report, we illustrate neural communication between the auriculotemporal and inferior alveolar nerves. On histological examination, the auriculotemporal nerve proximal to the communicating branch exhibited a greater number of nerve fascicles compared to the distal portion beyond the communication. Given that both nerves primarily serve sensory functions, this observation indicates that the sensory fibers potentially pass from the inferior alveolar nerve to the auriculotemporal nerve. Consequently, this observed neural connection could be implicated in referred pain from the anatomical territory of the inferior alveolar nerve (mandibular teeth) to regions innervated by the auriculotemporal nerve, including the temporomandibular joint, auricle, external auditory meatus, tympanic membrane, and the temporal skin. Further research is crucial to elucidate the prevalence and functional significance of neural communications within the infratemporal fossa.
